# THE INTERACTIVE ASSOCIATION OF EDUCATION AND NEIGHBORHOOD ENVIRONMENTS ON COGNITIVE DECLINE

**DOI:** 10.1093/geroni/igz038.102

**Published:** 2019-11-08

**Authors:** Michael H Esposito, Philippa Clarke, Ketlyne Sol, Jessica Finlay, Margaret Hicken, Suzanne Judd, Virginia Wadley

**Affiliations:** 1 University of Michigan, Ann Arbor, Michigan, United States; 2 University of Alabama at Birmingham, Birmingham, Alabama, United States

## Abstract

Education’s ambiguous association with cognitive decline may be due to unmeasured effect heterogeneity, including variation across environmental contexts. In areas with more social and physical resources, education may play less of a role in shaping cognitive trajectories. In areas with fewer resources, educational capital may be more important for slowing cognitive decline. Using multilevel models, this paper examines whether education’s impact on cognitive trajectories varies among neighborhoods defined by differential densities of social and physical resources. Findings suggest that education plays a consistent role in shaping cognition across contexts. Lower education is associated with lower cognitive function (High school vs College: b=-2.97; sd=0.16) and marginal differences in rates of decline (College vs High School: b=0.04; sd=0.03). However, these patterns are invariant across neighborhoods. Findings reiterate the importance of education for cognitive function in late life, and stimulate further research on other contextual factors that may affect rates of cognitive decline.

